# A comprehensive study of epitopes and immune reactivity among *Plasmodium* species

**DOI:** 10.1186/s12866-022-02480-7

**Published:** 2022-03-11

**Authors:** Meenu Kalkal, Amit Kalkal, Sandeep Kumar Dhanda, Emily Das, Veena Pande, Jyoti Das

**Affiliations:** 1 Parasite-Host Biology, National Institute of Malaria Research, Sector-8, Dwarka, New Delhi 110077 India; 2 Agriveda Private Limited, New Delhi, 110043 India; 3 Department of Oncology, St Jude Children’s Research Hospital, Memphis, 38105 USA; 4 Maulana Azad Medical College, New Delhi, 110002 India; 5 Department of Biotechnology, Kumaun University, Nanital, 263001 India

**Keywords:** *Plasmodium*, T cell epitopes, B cell epitopes, Interleukin-10 (IL-10), Interferon-γ (IFN-γ), MHC-binders, Antigenic proteins

## Abstract

**Background:**

Malaria is a life-threatening disease caused by protozoan parasite of genus *Plasmodium*. Various antigenic proteins of *Plasmodium* are considered as the major targets for the development of an effective vaccine. The aim of the current study was a comprehensive analysis of the experimentally validated epitopes of *Plasmodium* obtained from various immunoassays.

**Methods:**

*Plasmodium* species epitopes were prefetched from Immune Epitope Database (IEDB). Species specific classification of available epitopes was done for both human and murine malaria parasites. Further, these T cell and B cell epitopes along with MHC I/II binders of different *Plasmodium* species were examined to find out their capability to induce IFN-γ and IL-10 using IFNepitope and IL-10 Pred, respectively.

**Results:**

The species-specific classification of 6874 unique epitopes resulted in the selection of predominant human and murine *Plasmodium* species. Further, the attempt was made to analyse the immune reactivity of these epitopes for their ability to induce cytokines namely IFN-γ and IL-10. Total, 2775 epitopes were predicted to possess IFN-γ inducing ability, whereas 1275 epitopes were found to be involved in the induction of IL-10.

**Conclusions:**

This study facilitates the assessment of *Plasmodium* epitopes and associated proteins as a potential approach to design and develop an epitope-based vaccine. Moreover, the results highlight the epitope-based immunization in malaria to induce a protective immune response.

**Supplementary Information:**

The online version contains supplementary material available at 10.1186/s12866-022-02480-7.

## Background

Malaria, caused by the protozoan parasite of genus *Plasmodium* is a serious life-threatening infectious disease and mainly transmitted by female mosquito vector. According to recent World Malaria report (2021), there were an estimated 241 million cases of malaria around the world with approx. 0.4 million estimated deaths [1]. In humans, malaria is mainly caused by five different species: *Plasmodium falciparum*, *Plasmodium vivax*, *Plasmodium malariae Plasmodium ovale* and *Plasmodium knowlesi* Several recent reports suggest that *Plasmodium knowlesi* has an ability to infect humans along with simian malaria [2]. However, *P. falciparum* malaria parasite is accountable for most of the malaria-related demises with prevalence in the African content while *P. vivax* which has wider geographical range and considered to be less malignant than *falciparum*. Moreover, rodent malaria parasites such as *Plasmodium berghei*, *Plasmodium yoelii*, *Plasmodium chabaudi* and *Plasmodium vinckei* with different genetic background are being studied globally to understand immunology and other physiology of malaria [3].

Despite this species diversity, all *Plasmodium* species are known to share a similar basic life cycle. There is a clinically silent phase of infection in the liver while exponential growth of parasite occurs in blood stage [4]. With these similarities, still there is difference in severity of infection with different species, as *Plasmodium* parasite has developed several escape mechanisms by modifying its genome over the period of time. Therefore, due to multiple complexities involved at both host and parasite end, clinical representation of malaria may range from asymptomatic, uncomplicated to severe malaria. Additionally, different stages of the parasite life cycle are known to express a variety of surface proteins that activate different defence mechanisms of the host [5, 6]. This complexity of the parasite life cycle and malaria immunology presents a major challenge for vaccine development and eradication of malaria [7]. Till date, RTS,S is the only approved circumsporozoite (CS) protein based vaccine that has been developed for malaria and has shown limited efficacy against parasite infection [8]. Moreover, in-depth understanding of the immunological aspects involved during vaccination are not very sound. Therefore, there is need for a promising vaccine candidate that can provide long term sterile immunity to malaria. This is the reason various vaccine candidates have been explored in the past decade and many of them are in clinical trials [9, 10].

Whether human or non-human host, vaccines are known to activate immune system involving various immune mechanisms. In the present study, we have made a systematic attempt to understand the *Plasmodium* species-based epitopes and their ability to influence immune system. It is well believed that host immune response during *Plasmodium* infection is very complicated and requires interaction of parasite with RBCs during blood stage [11]. This blood stage infection is responsible for production of several cytokines by host immune system that are pro-inflammatory and anti-inflammatory in nature and a balance determines the outcome of disease towards susceptibility/resistance. Pro-inflammatory cytokines (TNF-α, IFN-γ, IL-1, IL-6 and IL-12) works against parasite by promoting the appropriate cell mediated and humoral immunity [12]. However, prolonged and elevated proinflammatory response may damage the host, here at this stage anti-inflammatory response plays a key role by limiting the pro-inflammatory response [13, 14]. Many aspects of this process need to be explored yet to understand how cytokines contribute to control of the immune response in malaria by detecting the differences between the protective and pathological modulatory effects.

In the light of the above discussion, this study was designed to assess various *Plasmodium* epitopes identified from different stages of parasite life cycle and subsequently these epitopes were characterised for their ability to induce the production of two selected cytokines, namely IFN-*γ* and IL-10. The balance of these cytokines known to determine the outcome of *Plasmodium* infection and thus it is important to investigate their underlying mechanism of action during infection. Interferon-*γ*, a key Th1-derived inflammatory cytokine is responsible for killing of intracellular parasites, however, overproduction may lead to tissue pathology [15, 16]. On the other hand, Interleukin-10 (IL-10), a Th2-derived anti-inflammatory cytokine well acknowledged to counterbalance inflammation and pathology associated with IFN- γ in both humans and experimental models [17]. IFN-γ, is a pro-inflammatory cytokine is a key mediator of inflammatory immune response and controls the infection at both liver and blood stage of *Plasmodium* infection [18]. IFN-γ is generated by NK, γδ, and NKT cells after liver and blood stages of malaria parasite infection and is responsible for mediating protective immunity against the parasite [19]. Once the adaptive immunity is established, CD4^+^ T cells become the major producer of IFN- γ which further activates cytotoxic T cells and performs a vital role in the cytotoxic killing of malaria parasite infected cells [20]. Additionally, IFN-γ stimulates isotype class-switching in B-cells that triggers the secretion of cytophilic antibodies [18]. These antibodies confer protective immunity by binding to the free malaria parasite and subsequently impede its ability to invade RBCs. Moreover, IFN-γ is the potent activator of macrophages and enhances their activities such as phagocytosis of infected erythrocytes as well as free parasites and production of inflammatory cytokines [21]. Various studies involving both human subjects as well as mice models suggested the importance of a fine balance between IFN-γ mediated protection and immunopathology associated with its aberrant expression [22–24]. Therefore, it is tempting to explore the dynamic IFN-γ response with malaria parasite infection and more efforts are required to understand the delicate balance of IFN-γ necessary for attaining best defence against parasite as well as minimizing the associated immunopathology at the same time [25].

Similarly, interleukin-10 (IL-10) is an important anti-inflammatory cytokine reported to have key role in infection by limiting the immune response to pathogen and thereby preventing the host. At first, it was described to be produced by Th2 cells to inhibit Th1 response. Now a days it is well known to be produced by various subsets of CD4^+^ and CD8^+^ T cells along with macrophages, B cells, NK cells, dendritic cells etc.[26]. Moreover, IL-10-producing Foxp3^+^Tbet^+^CD4^+^ regulatory T cells are considered an important source of IL-10 during malaria pathogenesis [27]. The role of IL-10 cytokine as an immunoregulator is well recognised in malaria pathogenesis by counteracting the effects of the proinflammatory cytokines mainly produced by Th1 cells. In addition to MHC-II, it is known to supresses inflammation by downregulating the expression of co-stimulatory molecules expressed on APCs [28]. Additionally, it is possible that enhanced production of IL-10 will be resulted in the exacerbation of disease by suppressing the parasite killing, considering that IL-10 may plays a unfavourable role during *P. falciparum* infection [29]. IL-10 is also a known inhibitor of the pro-inflammatory mediators released by monocytes/macrophages, resulting in LPS and IFN-*γ* induced inhibited secretion of TNF-*α*, IL-1*β*, IL-6, IL-8, G-CSF, and GM-CSF [30]. Moreover, results obtained using mouse models have suggested that IL-10 plays a protective role in the host during murine malaria infection [31–33].

## Results

### *Plasmodium* immune epitope database

*Plasmodium* (ID: 5820) genus in the IEDB database (an exclusive repository to access immunological data) resulted in 6874 unique epitopes that are positive or experimentally validated and comprising of both linear and discontinuous sequences. These epitopes are defined as peptides fragments that are immunogenic in nature and capable of producing a specific T cell or B cell immune response. Next, we retrieved all epitope sequences derived from a total of 14,816 immunoassays comprising of 3947 T cell assay, 8893 B cell assays and 1976 MHC ligand assays from approximately 800 studies. Further, only uniquely represented epitopes from all three assays were considered to determine their species-specific distribution by removing the repetitive epitope sequences.

### Species-specific distribution of *Plasmodium* epitopes

**Fig. 1:**
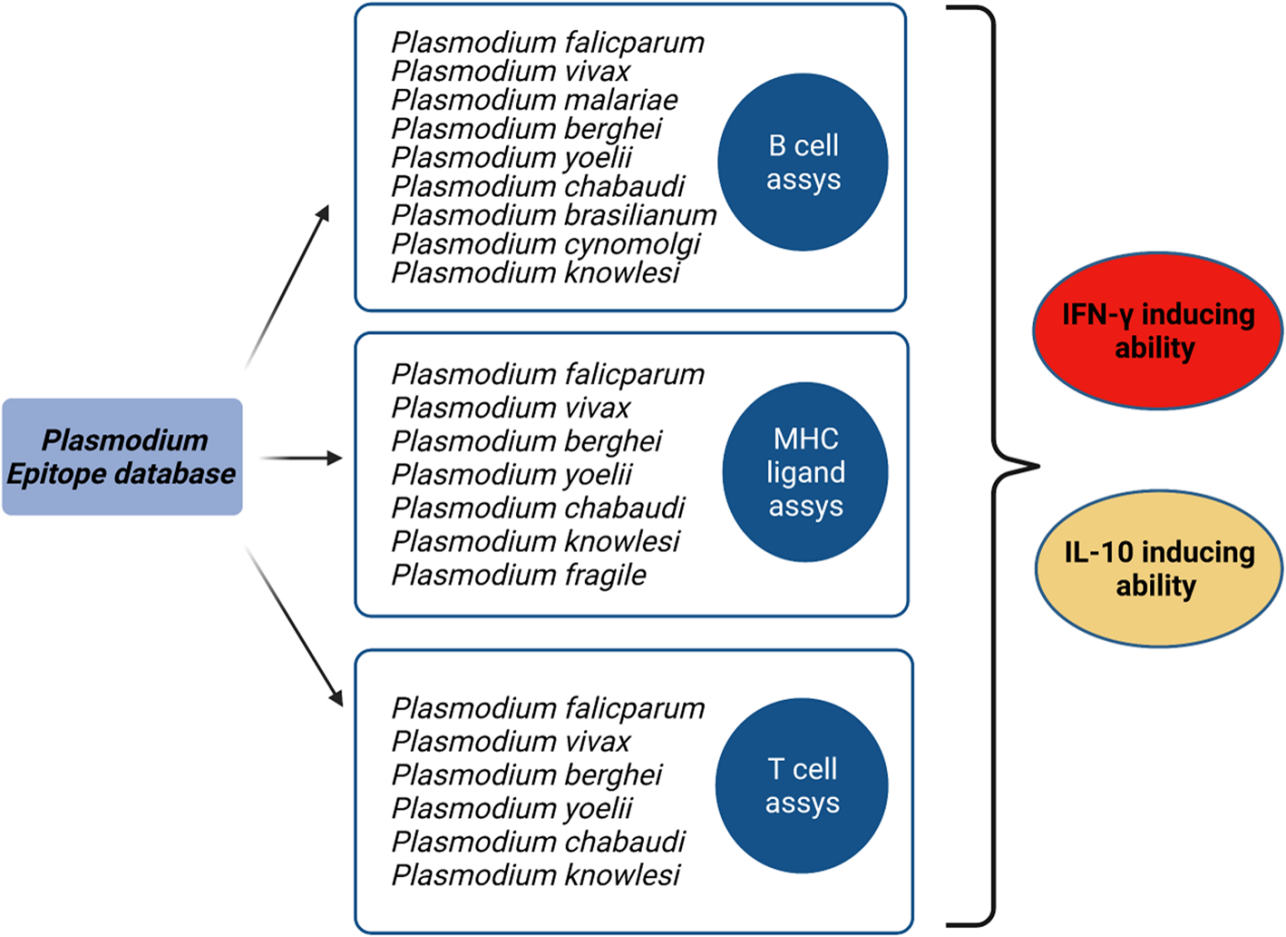
Flow chart of our study. Existing epitopes were retrieved from IEDB, enlisting B cell assays, MHC ligand assays and T cell assays. Immune reactivity of epitopes was predicted using the packages like IFNepitope, and IL-10 Pred

For species-specific classification of epitopes, both linear and discontinuous epitopes of *Plasmodium* genus obtained from T cell, B cell and MHC ligand assay were considered. As result, it was observed that these experimentally validated epitopes mainly belong to 9 different species of *Plasmodium* namely *P. falciparum, P. vivax, P. berghei, P. yoelii, P. chabaudi, P. brasilianum, P. cynomolgi, P. knowlesi* and *P. fragile* (Fig. 1). A detailed enumeration of epitopes distribution among all *Plasmodium* species is represented in Table 1. It was also observed that majority of these experimentally validated epitopes have been described for two most common human malaria parasites *P. falciparum and P. vivax.* Similarly*, P. berghei* and *P. yoelii* are the most prominent species found in studies involving murine models of malaria. Very few epitope sequences which belong to Genus *Plasmodium* but where species was not stated in data retrieved from IEDB were excluded from the further study. Therefore, based on high prevalence of epitopes, two most studied murine malaria species *P. berghei* and *P. yoelii* along with most prominent human malaria parasites namely *P. falciparum* and *P. vivax* were considered for further investigation.

**Table 1:** Species-specific classification of uniquely available *Plasmodium* Epitopes from different immunoassays

**Species**	**B cell epitopes**	**T cell epitopes**	**MHC I/II binders**
***Plasmodium falciparum***	4907	910	481
***Plasmodium vivax***	407	108	97
***Plasmodium malariae***	1	*	*
***Plasmodium berghei***	27	83	55
***Plasmodium yoelii***	36	61	23
***Plasmodium chabaudi***	6	41	1
***Plasmodium brasilianum***	2	*	*
***Plasmodium cynomolgi***	2	*	*
***Plasmodium knowlesi***	42	8	29
***Plasmodium fragile***	*	*	5
	5430	1211	691

^* ^Not known

### Epitopes distribution with different *Plasmodium* antigens

**Fig. 2:**
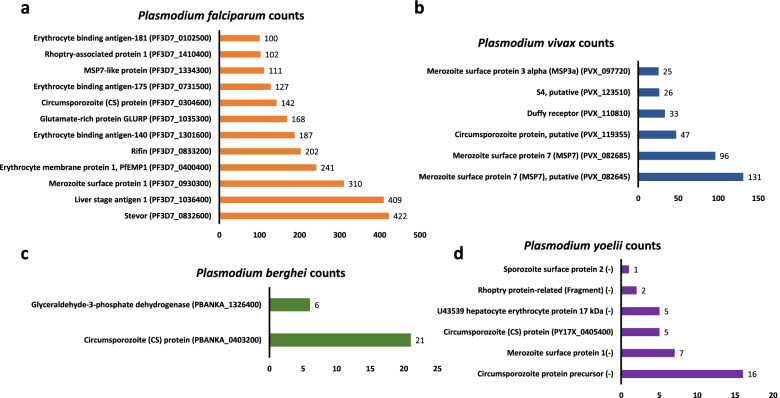
Prevalence of epitopes from B cell assays to their parent protein in *Plasmodium*

**Fig. 3:**
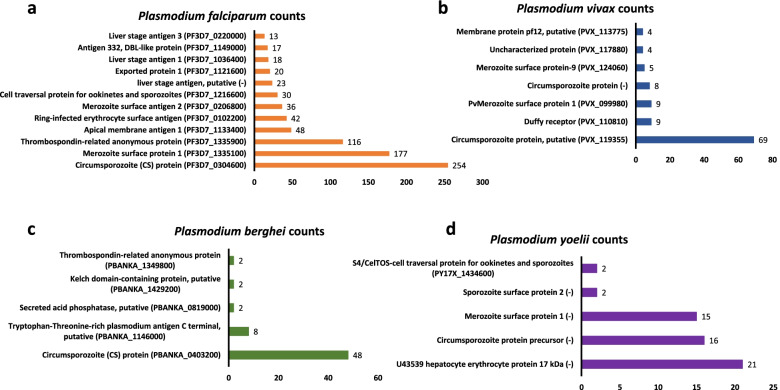
Prevalence of epitopes from T cell assays to their parent protein in *Plasmodium*

**Fig. 4:**
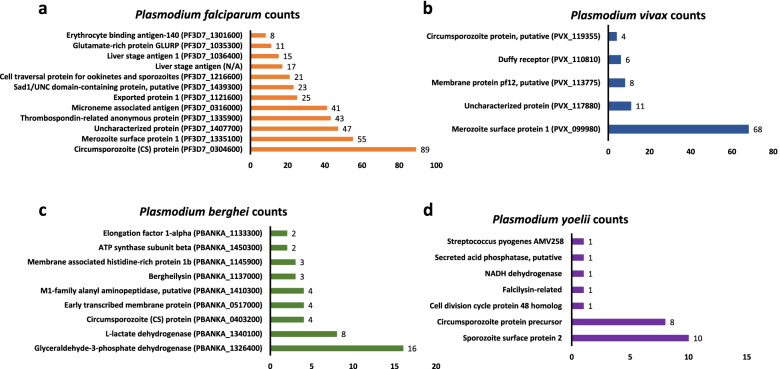
Prevalence of epitopes from MHC ligand assays to their parent protein in *Plasmodium*

After this initial species-specific sorting of epitopes, we checked the uniquely available T cell epitopes, B cell epitopes and MHC binders of four *Plasmodium* species to find out the major plasmodia antigens in each species. Prevalence or distribution of most of these major antigens associated with different life stages of four major *Plasmodium* parasite species are represented in Fig. 2 (B cell epitopes), Fig. 3 (T cell epitopes) and Fig. 4 (MHC I/II binders). Table S1 contains detailed information about number of epitopes associated with each antigen along with *Plasmodium* species information. Table S2 contains a list of epitope sequences and PlasmoDB ID of epitopes obtained from B cell assays, T cells assays along with MHC binders. It was observed that vastly represented epitope sequences belong to surface antigens from the hepatic and erythrocytic stages of malaria parasite. Circumsporozoite (CS) protein, putative circumsporozoite (CS) protein, thrombospondin-related anonymous protein (TRAP), liver stage antigen 1 (LSA 1), merozoite surface proteins (MSP 1, MSP2, MSP7), ring-infected erythrocyte surface antigen (RESA), Pv Merozoite surface protein 1 (PvMSP1), Duffy receptor etc. were the epitope dominant proteins. The assessment of these predominant antigenic proteins and epitopes from different stages of *Plasmodium* will be beneficial for the understanding of vaccine design. Additionally, with these top hit antigens, large number of other proteins from different developmental stages of *Plasmodium* have been investigated as vaccine candidates so far. However, antigen-specific host immune response is not completely known for these vaccine candidates. Therefore, a complete understanding of the immune response with different antigens needs to be explored. Though, importance of cytokines in mediating immune response during the various malaria parasite stages has been investigated or continues to be under investigation worldwide. Therefore, in current study these species-specific epitopes of both murine and human malaria parasites were processed to find out their cytokines inducing ability. And, to proceed further, we considered only linear peptide sequences by excluding conformational and other non-peptidic sequences. Additionally, epitopes sequences that were less than or equal to 30 residues were included in the current study for further analysis to find out their ability to induce IL-10 and IFN-γ cytokines using prediction softwares.

### IFN-γ inducing epitopes using IFNepitope

IFNepitope is a web server (https://webs.iiitd.edu.in/raghava/ifnepitope/) freely available to predict and design the epitopes having ability to induce release of interferon-γ cytokine. In current study B cell epitopes, T cell epitopes and MHC I/II binders of four most predominant species of *Plasmodium* were analysed for their IFN-γ inducing ability using IFNepitope server (Table 2, Table S3).

**Table 2:** Species-specific representation of number of IFN-γ inducing peptides/epitopes

**Epitopes**	***Plasmodium* Species Name**	**No. of IFN-γ inducing peptides**
**B cell epitopes**	*Plasmodium falciparum*	1386
	*Plasmodium vivax*	187
	*Plasmodium berghei*	17
	*Plasmodium yoelii*	30
**T cell epitopes**	*Plasmodium falciparum*	622
	*Plasmodium vivax*	84
	*Plasmodium berghei*	48
	*Plasmodium yoelii*	44
**MHC I/II binders**	*Plasmodium falciparum*	237
	*Plasmodium vivax*	82
	*Plasmodium berghei*	29
	*Plasmodium yoelii*	9

**Fig. 5:**
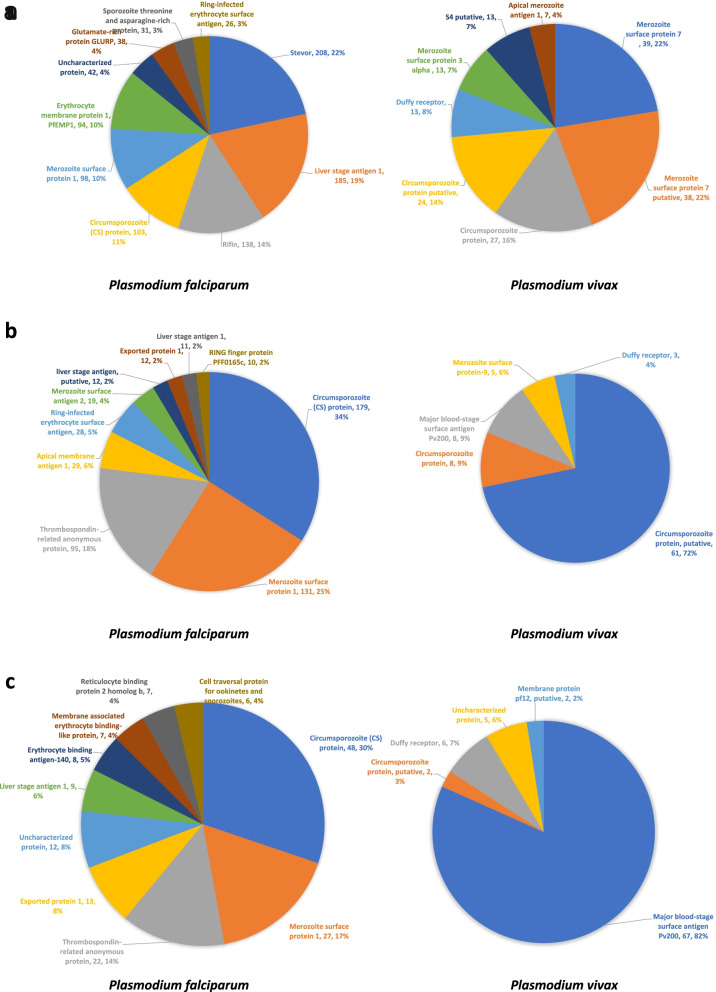
Prevalence of epitopes and their parent protein for IFN-γ inducing ability in four major species of *Plasmodium*

From B cell epitopes, among 2747 epitopes available for *Plasmodium falciparum*, 1386 were found to have IFN-γ inducing ability, and 187 IFN-γ inducing epitopes were found in *Plasmodium vivax* out of 382 unique epitopes available (Fig. 5a). This clearly shows that approximately 50% of the epitopes are having ability to induce an immune response through pro-inflammatory cytokine IFN-γ. It was also observed that vast majority of these epitopes positive for IFN- γ induction in *P. falciparum* were from proteins like stevor, liver stage antigen 1 (LSA1), rifin, circumsporozoite (CS) protein, Merozoite Surface Protein 1 (MSP1), Erythrocyte membrane protein 1(PfEMP1). Stevor is known to contribute to parasite-mediated pathology through rosetting, in so doing protects released merozoites from immune detection [34]. Other proteins like CS protein and MSP1 are also major *P. falciparum* blood-stage malaria vaccine candidates [35] involved in erythrocyte invasion [36]. Moreover, merozoite surface protein 7 (MSP7), putative merozoite surface protein 7, circumsporozoite (CS) protein, putative circumsporozoite protein and duffy receptor are the major *P. vivax* proteins which dominated for IFN- γ induction. Similarly, from T cell epitopes, for *P. falciparum*, 622 epitopes were IFN-γ inducing out of 885 epitopes, and 86 out of 107 epitopes were found to have IFN-γ inducing ability for *P. vivax* (Fig. 5b). Circumsporozoite protein (CS) protein, merozoite surface protein 1 (MSP1), thrombospondin-related anonymous protein (TRAP), apical membrane antigen 1 etc. are the proteins of *P. falciparum* where epitopes dominated for IFN- γ ability while epitopes of putative CS protein, CS protein and duffy receptor proteins of *P. vivax* were found to be dominated for IFN- γ induction. Additionally, when antigenic peptides from MHC binding assay were subjected IFNepitope for determining the prevalence of IFN- γ inducing epitopes, 237 epitopes were recorded positive for IFN-γ inducing ability out of 479 available peptides of *P. falciparum*, and 82 were found positive out of 97 epitope sequences for *P. vivax*. It was also observed that CS protein, MSP1 are the proteins of *P. falciparum* that were having maximum number of positive epitopes for IFN-γ Merozoite surface protein 1 (PvMSP1) and duffy receptor are top proteins of *P. vivax* with the highest number of IFN- γ inducing epitopes obtained from MHC binding assays (Fig. 5c).

In addition, IFN-γ inducing ability in murine malaria parasites namely *P. berghei* and *P. yoelii* was also checked for experimentally validated available epitopes. It was observed that 17 out of 25 unique epitope peptide sequences of two main proteins CS protein and Glyceraldehyde-3-phosphate dehydrogenase belonging to *P. berghei* available from B cell assays were found to have IFN-γ inducing ability, and 30 out of 36 were positive for IFN-γ with *P. yoelii* proteins mainly circumsporozoite protein precursor, Merozoite surface protein 1. Similarly, for *P. berghei* epitopes available from T cell assays, 48 out of 82 were having IFN-γ inducing ability and 44 out of 60 epitopes from *P. yoelii* were positive for IFN-γ. These epitopes were mainly from CS protein of *P. berghei* and MSP-1, U43539 hepatocyte erythrocyte protein 17 kDa (*Py*HEP17) and circumsporozoite protein precursor of *P. yoelii*. Also, epitopes available from MHC binding assays were checked for IFN-γ prediction. It was found that among 55 epitopes of *P. berghei*, 29 were positive for IFN-γ prediction and 9 out of 23 were found to have IFN-γ inducing ability for *P. yoelii*. Major proteins to which these epitopes from MHC binding assay belongs are L-lactate dehydrogenase, glyceraldehyde-3-phosphate dehydrogenase, CS protein, circumsporozoite protein precursor and sporozoite surface protein 2.

### IL-10 inducing epitopes using IL-10 pred

Next, IL-10 pred analysis was performed using IL-10 pred (https://webs.iiitd.edu.in/raghava/il10pred/) web server, for determination of IL-10 inducer and IL-10 non-inducer antigenic peptides. B cell, T cells and MHC I/II binders of four major species of *Plasmodium* were checked for IL-10 inducing ability using IL-10 pred (Table 3, Table S4).

**Table 3:** Species specific representation of number of IL-10 inducing peptides/epitopes

**Epitopes**	***Plasmodium* Species name**	**No. of IL-10 inducing peptides**
**B cell epitopes**	*Plasmodium falciparum*	647
	*Plasmodium vivax*	74
	*Plasmodium berghei*	4
	*Plasmodium yoelii*	5
**T cell epitopes**	*Plasmodium falciparum*	302
	*Plasmodium vivax*	29
	*Plasmodium berghei*	14
	*Plasmodium yoelii*	13
**MHC I/II binders**	*Plasmodium falciparum*	128
	*Plasmodium vivax*	51
	*Plasmodium berghei*	6
	*Plasmodium yoelii*	2

**Fig. 6:**
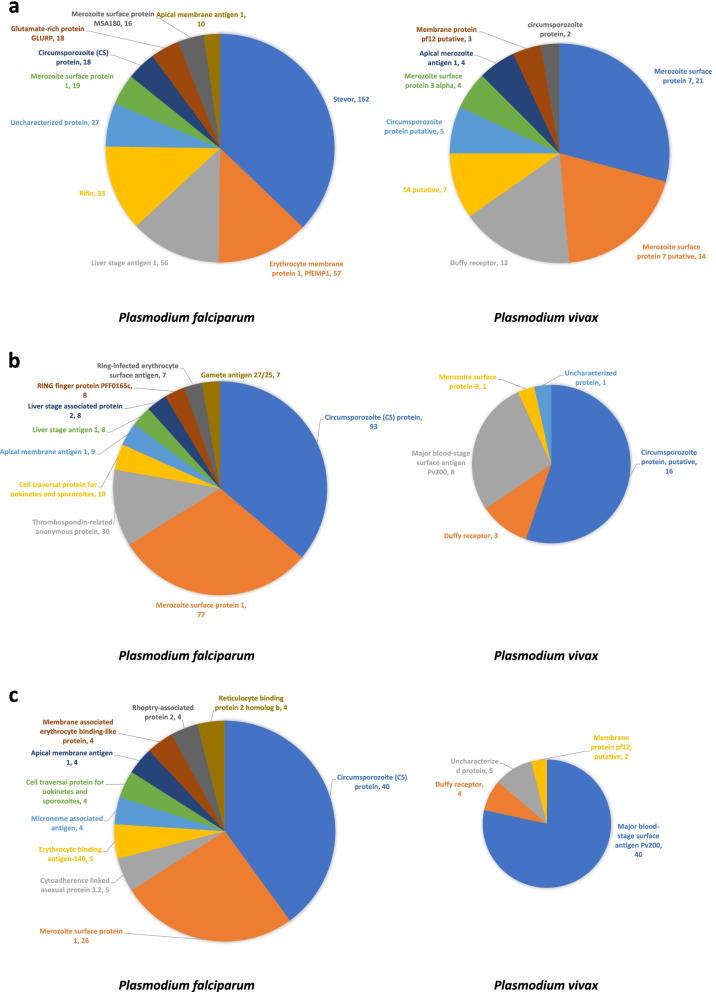
Prevalence of epitopes and their parent protein for IL-10 inducing ability in four major species of *Plasmodium*

From B cell epitopes, among 2747 *P. falciparum* epitopes, 647 were found to have IL-10 inducing ability and a total of 74 epitopes from 382 unique epitopes of *P. vivax* were found to have IL-10 inducing ability (Fig. 6a). Interestingly, the number of IL-10 inducer peptides identified mainly were for proteins like stevor, erythrocyte membrane protein 1 (PfEMP1), liver stage antigen 1 (LSA-1), rifin etc. for *P. falciparum* while MSP-7, MSP-7 putative and duffy receptor were major proteins for *P. vivax*. Likewise, from T cell epitopes, for *P. falciparum*, 302 epitopes were IL-10 inducing out of 885 epitopes, and 29 from a total number of 107 epitopes were found to have IL-10 inducing ability for *P. vivax* (Fig. 6b). Moreover, most of these epitopes belong to circumsporozoite protein of *P. falciparum* and putative circumsporozoite protein of *P. vivax*. Additionally, when antigenic peptides or epitopes from MHC assay were processed on IL-10 pred, 128 epitopes of *P. falciparum* were positive for IL-10 inducing ability out of 479 uniquely available peptides, and 51 were found positive out of 97 epitope sequences for *P. vivax* (Fig. 6c). The maximum number of antigenic peptides for *P. falciparum* were found belonging to circumsporozoite protein and Pv200, a major blood surface antigen of *P. vivax* dominated for IL-10 inducing ability.

We also analysed IL-10 inducing epitopes in murine malaria parasites namely *P. berghei* and *P. yoelii*. It was observed that 4 out of 25 unique antigenic peptide sequences available from B cell assays with *P. berghei* were having IL-10 inducing ability belonging to two major proteins namely circumsporozoite protein and glyceraldehyde-3-phosphate dehydrogenase, and 5 epitopes out of 36 B-cell epitopes were found to have IL-10 inducing ability with *P. yoelii* belonging to circumsporozoite protein precursor, circumsporozoite (CS) protein. Similarly, for *P. berghei* T cell epitopes, 14 out of 82 epitopes were identified with IL-10 inducing ability and 13 out of 60 epitopes from *P. yoelii* were positive for IL-10 induction. Most of these T cell epitopes are represented by CS protein, circumsporozoite protein precursor, merozoite surface protein 1. Along with T cell and B cell epitopes, epitopes available from MHC binding assays were also subjected for predicting IL-10 inducing ability. It was found that among 55 epitopes of *P. berghei*, 6 were positive for IL-10 prediction and 2 from a total of 23 epitopes were found to have IL-10 inducing ability for *P. yoelii*. These epitopes with IL-10 inducing ability were representation of three major proteins like circumsporozoite protein precursor of *P. yoelii* and L-lactate dehydrogenase and ATP synthase beta subunit of *P. berghei*.

### IFN-γ and IL-10 inducing epitopes from IEDB database

In addition to prediction of IFN-γ and IL-10 inducing ability of epitopes by IFNepitope and IL-10 Pred respectively, we also assessed the experimentally validated IFN-γ and IL-10 inducing epitopes available from T cell assays in the IEDB database. Epitopes from four major species including human and murine malaria parasites namely *Plasmodium falciparum, Plasmodium vivax, Plasmodium berghei, Plasmodium yoelii* were included for next analysis. These experimentally validated epitopes were cross-checked for their presence in epitopes predicted with IFN-epitope and IL-10 Pred. Details of these epitopes is provided in Supplementary file S5 with common epitopes (predicted by software and experimentally validated) highlighted in yellow colour.

It was observed that almost all experimentally validated epitopes positive for IFN-γ inducing ability from IEDB database are similar epitopes predicted with software. Very few epitopes among four species from IEDB database that are experimentally positive for IFN-γ and were not predicted by software due to long epitope length are also listed in Table S5 (Green in colour) along with their sequences. It was also observed that vast majority of these epitopes experimentally positive for IFN- γ induction belong to *P. falciparum* (529 epitopes from a total of 654 epitopes). Moreover, it was noticed that most of these epitopes belongs to proteins like circumsporozoite (CS) protein, Merozoite Surface Protein 1 (MSP1) of *Plasmodium falciparum*.

Similarly, all experimentally validated epitopes with IL-10 inducing ability form IEDB database were found same as predicted by IL-10 pred except one epitope that belong to merozoite surface protein 3 (MSP3). Moreover, we observed that number of epitopes predicted by software are more than number of epitopes experimentally validated. Only 68 epitopes from IEDB database that are positive for IL-10 induction are known as compared to 358 epitopes predicted by IL-10 Pred. Additionally, we observed that most of these experimentally validated epitopes belongs to *P. falciparum* (53 out of 68 epitopes) and *P. vivax* (12 out of 68 epitopes). Only 3 epitopes belonging to murine malaria parasite *P. berghei* are positive for IL-10 induction. Further observations indicated that most of these epitopes belongs to merozoite surface protein 1 (MSP 1), thrombospondin-related anonymous protein (TRAP) and circumsporozoite (CS) protein of *Plasmodium falciparum*.

## Discussion

Various antigenic proteins specific to different stages of *Plasmodium* are considered as the major targets for the development of an effective vaccine [37]. Moreover, many epitopes associated with these antigenic proteins have been examined for ability to induce immune system through various *in-vivo* and *in-vitro* assays, a critical step required in the development of epitope-driven vaccines to determine the immune response post-immunization.

Species specific classification of *Plasmodium* epitopes in current study provided us evidence about predominant murine and human malaria parasites. Additionally, number of epitopes in each species explored so far were recorded. Although, it is also important to mention that the predominant status of a given *Plasmodium* epitope in the immune epitope data is not the true representation as many genetic and experimental factors may attribute to immunodominance of an epitope [5]. Further observations of these epitopes and associated proteins of four major species provided us deep insights about major antigenic proteins and their prevalence among different species. Circumsporozoite (CS) protein, most prominent sporozoite surface antigen of malaria parasite [38] was observed to present in almost all species of *Plasmodium* whether human or non-human. It is an important vaccine candidate and has been investigated throughout the world in various studies for its efficacy [39]. Thrombospondin-related anonymous protein (TRAP) is another frequently investigated *Plasmodium* protein essential for sporozoite motility and liver cell invasion [40]. Therefore, TRAP epitopes may serve as vaccine candidates for sporozoite-based malaria vaccines. *P. falciparum* liver-stage antigen 1 (LSA-1) exact function is not clear to date, however, it is thought to have a role in liver schizogony and release of merozoites [41]. It is well conserved protein and contains potent B and T cell determinants [42] with ability to induce immune response making it an important vaccine candidate [43]. Similarly, merozoite surface protein 1 (MSP1) is another antigen that has been widely considered as a vaccine candidate and abundantly represented among all GPI-anchored surface proteins [44]. Various investigations involving the MSP-1 mediated immune response have suggested its high immunogenicity under natural infections with *P. falciparum* and *P. vivax* [45–49]. Moreover, MSP family is most studied as a vaccine candidate against malaria. Major blood surface antigen Pv200 for *P. vivax* is an analogue of MSP-1 from *P. falciparum* [50]. Many murine studies have indicated the high antigenicity of Pv200 protein [50, 51] and a high antibody titers against the protein have been observed in naturally infected individuals[51, 52]. Another *P. falciparum* protein, ring-infected erythrocyte surface antigen (RESA) located at the membrane of erythrocytes infected with ring-stage parasites, is also a major vaccine candidate against *P. falciparum* and has been studied widely [53]. A large number of other proteins have been identified along with these major vaccine candidates have been identified from epitope data. Further analysis with these epitopes and their proteins for their ability to induce IL-10 and IFN-γ, two major cytokines that are known to determine the outcome of *Plasmodium* infection [18, 54] provided valuable information about IL-10 and IFN-γ inducing peptides. In malaria, IFN-γ mediated protection has been demonstrated in murine studies and natural infection against pre-erythrocytic parasite infection [55, 56]. Also, IFN-γ associated responses against pre-erythrocytic or blood-stage antigens have shown protection from re-infection in African children [57]. Multiple evidence obtained from circumsporozoite (CS) protein based malaria vaccine, RTS,S, mediated protection also seems to be connected with sustained production of IFN-γ [58, 59]. Therefore, for a successful malaria strategy, it is important to consider IFN- γ response with other vaccine candidates to get a deeper understanding of the immune response. In the current study, high number of epitopes were found to have IFN-γ inducing ability in each species. Similarly, a vast majority of IL-10 inducing peptides (approx. 26%) in both human and murine *Plasmodium* species were identified. It was apprehended that most of these epitopes belong to parasite proteins like CS protein, stevor, merozoite surface protein 1 and erythrocyte membrane protein 1 (EMP 1) from both liver and blood stages of parasite indicating a broader immune response. IL-10 concentration has been interrelated with parasite density implying that IL-10 has an important immunoregulatory role, pronounced in both experimental and human models of malaria [27, 60–62]. Additionally, many studies involving *P. falciparum* natural infections endorses the protective role of IL-10 [63, 64] and suggests to further investigate underlying protective mechanisms with different proteins.

Conclusively, it was observed that 50% of the epitopes from each species hold IFN-γ inducing ability. Similarly, approx. 26% epitopes of unique epitopes from each species of *Plasmodium* were predicted to possess IL-10 inducing potential. Several studies have demonstrated the protective role of IFN-γ and IL-10 against a number of plasmodial antigenic proteins like LSA-1, MSP-1, CS protein, erythrocyte membrane protein (EMP) etc. [22, 24, 63, 65–68]. Additionally, IFN-γ/IL-10 co-producing cells also have been described as a dominant population of CD4^+^ T cells in acute malaria infection with malaria parasites [69]. This analysis will help in characterization of various epitopes based on immune response that may be pro-inflammatory or anti-inflammatory at early stage of infection. With this knowledge and new emerging approaches, the information generated through this study will be helpful in improving the success rate of epitopes-based immunome-derived vaccines (IDVs).

## Conclusions

This comprehensive analysis provides a wider depiction of malaria-related epitope data of both murine and human *Plasmodium* species. It also helps in identification of epitopes and associated antigens known to express at different stages of the parasite life cycle. The current study also provides a comprehensive as well stringent analysis of *Plasmodium* epitopes and proteins associated with their ability to modulate immune response through induction of cytokines like IL-10 and IFN-γ. Gaining insights into the epitopes and their ability to induce some important cytokines paves the ways for assessment of *Plasmodium* proteins as candidate for design and development of an epitope-based vaccine in future. The vaccine development based on synthetic peptides is already proved as successful strategy in many infectious diseases and is well established in cancer field. In malaria, the efforts made in current study may enhance vaccine development and understanding the pathogenesis caused by malaria parasite. However, it is also possible that there may be differences in sequence of some proteins when host is different. Therefore, to have a comprehensive overview of *Plasmodium* epitopes and their ability to modulate immune response, we included both murine and human *Plasmodium* species in the current study.

These observations in the present study suggested that circumsporozoite protein predominates along with other proteins and is exclusively expressed in all species of *Plasmodium* parasite and are in accordance with its promising candidate for malaria vaccine development. RTS,S/AS01 (RTS,S) vaccine developed for malaria is well known first human malaria vaccine based on Circumsporozoite (CS) protein. Progress with RTS,S represents a historic milestone, but its partial efficacy leaves room for further improvement. Meanwhile, novel malaria vaccine candidate clinical development has continued apace, being new R21/Matrix-M under trial is again a circumsporozoite protein-based vaccine [70]. Additionally, based on the results from the current study, some other top antigenic proteins and their epitopes may advance our understanding to explore them as foremost vaccine candidates.

## Methods

### Retrieval and preliminary analysis existing epitope data of *Plasmodium*

The Immune Epitope Database and Analysis Resource (IEDB; IEDB.org) is a freely available data resource funded by the National Institute for Allergy and Infectious Diseases (NIAID). It is a repository of experimental data on antibody and T cell epitopes with context of four broad disease categories: Infectious diseases, allergy, autoimmunity and transplantation. It also offers multiple tools for epitopes prediction and analysis [71]. Epitopes of all *Plasmodium* species were prefetched from IEDB using *Plasmodium* (ID: 5820) by considering all three search parameters available such as T and B cell epitopes along with MHC binders. Also, the MHC restriction pane was selected for any MHC available and all host parameter was selected to include all available host whether human, or non-human. Data prefetched with above mentioned search criteria included epitopes from different species such as *Plasmodium falciparum, Plasmodium vivax, Plasmodium malariae, Plasmodium knowlesi, Plasmodium berghei, Plasmodium chabaudi, Plasmodium yoelii, Plasmodium brasilianum, Plasmodium cynomolgi*. This information was further processed for preliminary analysis.

Initially all three files encompassing experimentally validated epitopes from T, B and MHC ligand assays were downloaded including detailed information about epitope sequences, epitope ID, parent species and parent protein of epitopes. These epitopes were subjected to classification for each available species of *Plasmodium* whether belong to human or animal (murine/ non-human primates). Next, each species was checked for the availability of all antigenic proteins known so far. Further, unique antigens or parent proteins along with their prevalence in each *Plasmodium* species were recorded for further analysis.

### Arrangement and sorting of species-specific peptide sequences

Peptide sequences are considered as a key input for prediction of their ability to induce different cytokines. First, all non-linear, discontinuous peptides and other non-peptidic sequences were excluded for further analysis. Next, the peptide sequences which were reported more than once in literature were considered as one during fasta file generation by removing identical sequences. Moreover, peptide sequences of more than 29 amino acids long were excluded for further study due to their incompatibility with epitope prediction software. Next to streamline further analysis, T cell and B cell epitopes along with MHC I/II binders of different *Plasmodium* species were used individually for fasta files generation. Further, these individual species fasta files were processed to find out their capability to induce two major cytokines (IFN-γ and IL-10).

### IL-10 Pred and IFNepitope analysis

Epitopes belonging to the two most important parasite species *P. falciparum* and *P. vivax* that are responsible for human malaria were subjected to publicly available web based servers namely IFNepitope [72] and IL10 pred [73], to find their ability to induce IFN-γ and IL-10 cytokines, respectively. Moreover, epitopes or peptide sequences of murine models of malaria namely *P. berghei* and *P. vivax* were also examined for their potential to induce IL-10 and IFN-γ using the same web-based servers.

IFNepitope server allows users to predict the IFN-γ inducing epitopes from a set of peptide sequences or library of peptides. Therefore, this server was used to predict IFN-γ inducing *Plasmodium* epitopes for each species. Search model used for prediction was SVM (support vector machine) motif hybrid which provides results after combining the SVM based model with motifs contributing to IFN-γ induction. The model performs better compared with SVM model and motif based model separately. Similarly, IL-10 Pred a web server was used, which is freely available that allows users to discover, design and mapping of peptides with capability to induce IL-10 cytokine. *Plasmodium* epitopes for determining their IL-10 inducing ability were subjected IL-10 pred with SVM based prediction model. In case of IL-10 Pred we used the default cut off values as recommended by developers. Here in addition to predicted values of IL-10 induction, we can also get the different physiochemical parameters assigned based on amino acid index database.

## Supplementary Information

**Additional file 1.**

**Additional file 2.**

**Additional file 3.**

**Additional file 4.**

**Additional file 5.**

## Data Availability

The data used analysed in the current study are available from the corresponding author on reasonable request.
